# Comparative efficacy of topical tetraVisc versus lidocaine gel in cataract surgery

**DOI:** 10.1186/1471-2415-9-7

**Published:** 2009-08-17

**Authors:** KV Chalam, Ravi K Murthy, Swati Agarwal, Shailesh K Gupta, Sandeep Grover

**Affiliations:** 1 Department of Ophthalmology, University of Florida College of Medicine, Jacksonville, Florida, USA

## Abstract

**Background:**

To compare the clinical efficacy of lidocaine 2% with tetracaine 0.5% for cataract surgery.

**Methods:**

In a randomized, multi-surgeon, controlled clinical trial,122 consecutive cataract cases eligible for topical anesthesia, were randomly assigned to receive lidocaine 2% gel (1 ml) or tetracaine solution 0.5% (TetraVisc, 0.5 ml) before clear corneal phacoemulsification. Main outcome measure was visual analog scale (0 to 10), which was used to measure intra-operative pain. Secondary outcome measures included patients' discomfort due to tissue manipulation and surgeon graded patients' cooperation. Duration of surgery and intra-operative complications were also recorded.

**Results:**

The mean age in TetraVisc (TV) group was 70.4 years and in the lidocaine gel group (LG) it was 70.6 years (p = 0.89). Patient reported mean intra-operative pain scores by visual analog scale were 0.70 ± 0.31 in TV group and 1.8 ± 0.4 in LG group (*P *< 0.001). Mean patient cooperation was also marginally better in the TV group (8.3 ± 0.3) compared to LG group (8.4 ± 0.6) (P = 0.25). 96% of patients in TV group showed intra-operative corneal clarity compared to 91% in LG group. TV group had less (1 out of 61 patients, 1.6%) intra-operative complications than LG group (3 out of 61 patients, 4.8%). No anesthesia related complications were noted in either group

**Conclusion:**

Topical TetraVisc solution was superior to lidocaine 2% gel for pain control in patients undergoing clear corneal phacoemulsification. Lidocaine 2% gel is similar to TetraVisc in patient comfort and surgeon satisfaction.

**Trial Registration:**

**Clinical trials number**: ISRCTN78374774

## Background

Cataract surgery is the most frequently performed surgical procedure in USA. Common forms of local anesthesia for cataract surgery include injection techniques (retrobulbar, peribulbar, subconjunctival, sub-Tenon's) and topical anesthesia [1-7]. Topical anesthesia has gained popularity in recent years, as it has been shown to be safe and effective method of anesthesia. The main advantages of using topical over injectable anesthetics are avoidance of risks and complications associated with injections, improved patient comfort with faster recovery, and lack of pain during administration [6,7]. Topical anesthetics can be used as drops, gel or intracameral injection in cataract surgery. Gel anesthesia with lidocaine 2% has shown higher intracameral levels of lidocaine, better analgesia, better patient cooperation and less need for intraoperative analgesia over lidocaine 4% topical drops [8]. Prolonged contact time and lubricating effectof gel anesthesia leads to increased efficacy and avoids the need for frequent corneal wetting. The disadvantages for the lidocaine gel are that its use demands several doses of administration, has short anesthetic effect, and at times obscures the view during capsulorrhexis or at the end of cataract procedure [9].

TetraVisc hydrochloride 0.5%, a new viscous topical agent induces rapid anesthesia as it disperses across the cornea rapidly and immediately and offers an extended period of anesthetization due to prolonged contact time [10].

Using a 10-point visual analog pain scale, we evaluated the efficacy of TetraVisc 0.5% solution (TV) and lidocaine gel 2% (LG) in a randomized study in patients undergoing phacoemulsification with intraocular lens implantation.

## Methods

The study was approved by Institutional Review Board/Ethics Committee of University of Florida. 122 patients scheduled for elective cataract surgery and eligible for topical anesthesia were included in the study. Exclusion criteria were unwillingness to have topical anesthesia, high anxiety, dementia or mental instability, deafness, movement disorders, hyperanxiety, and inability to complete the visual analog scale (VAS) of pain line (for example, confusion, communication barriers, visual impairment) and any reported allergy to lidocaine or tetracaine. All patients were recruited between January 2006 and November 2006.

The patients were randomized by block randomization (randomly assigned by computer generated numbers) to receive either TetraVisc (Tetracaine hydrochloride 0.5%, OCuSOFT) solution or lidocaine gel (Xylocaine 2% jelly, Astra) consecutively. Five doses of TV or LG were applied every 5 minutes, 20 minutes prior to surgery. No intravenous or oral sedation was used as pre-medication.

### Surgical Procedure

Ofloxacin was administered by the patient for 3 days preoperatively in the eye to be operated. Mydriasis was obtained preoperatively with phenylephrine hydrochloride, 2.5% (EyePhrine, Eye Supply, USA) and tropicamide, 1% (EyeMed, Eye Supply, USA) eye drops. In the operating room, baseline pulse and peripheral blood pressure were taken. The lid and periorbital skin were cleaned and the conjunctival cul-de-sac was irrigated with povidone iodide solution. The eye was then draped, and an open-wire speculum was placed. Before the surgeon sat at the operating microscope and began the procedure, the eye was irrigated with balanced salt solution to eliminate lidocaine gel or other anesthetic remnants. The surgical procedure was carried out as a clear corneal phacoemulsification using a superior incision by the phaco chop technique. Balanced salt solution was used for automated irrigation-aspiration. Foldable intraocular lenses (IOL) were implanted. During the surgery, the surgeon was in constant communication with the patients and frequently asked them if they wished to have additional anesthesia. If breakthrough pain occurred in either group during surgery, supplemental topical anesthesia was administered.

#### Primary Outcome Measures

After the completion of surgery, the patients were accompanied to the recovery room and were asked to grade intra-operative pain using a 0 to 10 visual analog scale (VAS) within 10 minutes of completion of surgery. The patient and independent observer were masked to the anesthesia used.

#### Secondary Outcome Measures

An independent observer also collected surgeons' responses to complete a ten point satisfaction scale immediately after each surgery rating the overall surgical experience. The surgeons were requested to consider not only a subjective impression of patient comfort and ease of surgery but also the presence of inadvertent eye movements, patient reported painful sensations (including pain during the administration of the anesthetic), lid squeezing, and intra-operative complications when providing an overall score. The final score was a general estimate considering all these variables. The scale used rated the surgical experience as 0–10 where 0 is poor cooperation and 10 is excellent cooperation. The need for supplemental anesthesia was recorded. Further information recorded included: patient demographics, operative technique, quantity and type of sedative administered, and volume of anesthetic used for supplemental anesthesia. The power of this study was 92% using a sample size of 61 patients to find a difference in VAS score of 1.0 with a standard deviation (SD) of 0.5 and a type 1 error of 0.05 between groups. For the surgeon satisfaction scale the power was 82% to find a difference between groups of 0.5 with SD of 0.65 and a sample size of 61 patients and a 0.05 type 1 error.

Intraoperative complications and corneal clarity were recorded at the end of surgery. Postoperative pain was graded again using a 10-point visual analog scale on the first postoperative day approximately 24 hours later. Student T-test was used to compare both groups. (Graphprizm, CA)

## Results

The results of the study are summarized in Table 1. One hundred twenty two eyes of 122 patients were operated for cataract in the study: 61 patients were randomly assigned to the TV group and 61 to the LG group. In TV group there were 25 males and 36 females and in LG group there were 28 males and 33 females. The mean age in TV group was 70.4 ± 4.1 (mean ± SD) years and in LG group was 70.6 ± 10.5 (mean ± SD) years. There was no significant difference in age between the two groups (p = 0.89). There were no anesthesia-related complications.

**Table 1:** Patient characteristics and results of efficacy of anesthesia in the TetraVisc and Lidocaine gel group

	TetraVisc group (Mean +/- SD*)	**Lidocaine gel group (Mean +/- SD)**	**P value**
**Age (in years, mean **± **SD)**	70.4 ± 4.1	70.6 ± 10.5	0.98

**Visual analog pain scores**	0.7 ± 0.31	1.8 ± 0.4	< 0.001

**Patient Cooperation**	8.3 ± 0.3	8.4 ± 0.6	0.24

**Clear Corneal clarity**	59/61(97%)	55/61(90%)	0.16

**Intra-operative complications**	1/61 (1.6%)	3/61(4.8%)	0.22

* SD: Standard deviation

### Primary Outcome Measures

Intraoperative pain scores by VAS were 0.7 ± 0.31 (mean + SD) in the TV group and 1.8 ± 0.4 (mean + SD) in the LG group. This difference was statistically significant (*P *< 0.001).

### Secondary Outcome Measures

Patient cooperation, as graded by the surgeon, was 8.3 ± 0.3 (mean ± SD) in TV group and 8.4 ± 0.6 (mean ± SD) in the LG group (p = 0.25).

Intraoperative corneal clarity was good in 59 of 61 patients (97%) in the TV group and in 55 of 61 patients (90%) in the LG group (p = 0.16).

The mean duration of surgery was 13.1 ± 2.7 minutes overall with mean of 13.4 ± 2.3, 12.4 ± 3.4 and 13.7 ± 2.1 minutes for the 3 surgeons who were involved in the study (p = 0.07). Mean VAS scores for the 3 surgeons were 8.20 ± 0.5, 8.1 ± 0.4 and 8.3 ± 0.4 respectively (p = 0.12).

1 out of 61 patients (1.6%) had difficulty in IOL implantation (Surgeon could not dial in the trailing haptic in a single attempt) in TV group compared to 3 out of 61 (4.8%) patients in LG group. All other minor complications (tearing of capsulorrhexis in one patient in TV group,) were managed uneventfully during surgery.

## Discussion

TetraVisc 0.5% is a recently introduced viscous topical anesthetic used for ocular surgery. It disperses across the cornea rapidly and offers an extended period of anesthesia making it superior to topical gels. Previous reported studies have shown topical tetracaine solution 0.5% with intracameral lidocaine to be safe and effective in cataract surgery [10].

In our study, mean intra-operative pain as measured by visual analog score was less in the TV group than LG group, indicating that topical TetraVisc was superior to lidocaine 2% gel for pain control in patients undergoing clear corneal phacoemulsification. Amiel et al have compared the anesthetic effect of the two drugs and concluded that their effects were similar [11]. But the limitation of their study was absence of standardization of the pain quality using the VAS.

Specific factors evaluated during this study included patient comfort and the surgeon's subjective impression of patient comfort and ease of surgery. Visual analog scale is a simple and frequently used method for assessment of intensity of pain [12]. In clinical practice the percentage of pain relief, assessed by VAS, is often considered as a measure of the efficacy of treatment and VAS score correlates well with acute pain levels [12,13]. Myles et al have shown that the VAS has properties consistent with a linear scale in patients with acute mild-to-moderate pain implying that a change in the VAS score represents a relativechange in the magnitude of pain sensation [13]. Due to the linear nature of the scale, studies have supported the use of parametric tests for analyzing VAS scores [13,14]. Patient comfort was superior in TV group compared to LG group (*P *< 0.001). No difference was observed between the two groups in terms of surgeons' satisfaction and patient cooperation.

Studies have not shown any corneal endothelial toxicity with lidocaine 2% gel both in animal and humans [15]. In our series, intra-operative corneal clarity was better in the TV group compared to the LG group. 97% had clear cornea in TV group as compared to 90% in LG group. Two eyes (3%) in TV group and 6 eyes (10%) in LG group showed mild intra-operative central corneal edema. This could be either due to hydration, variability of operative technique and operative time or lidocaine toxicity.

One of the limitations of the study was non-masked design, and patients were operated by three different surgeons. Inter-surgeon variability in technique and duration of surgery can influence the outcome of the study, even though this has not been the case in our report.

## Conclusion

In summary, both topical TetraVisc 0.5% and lidocaine 2% gel used independently provide good surgical anesthesia and patient comfort during ocular surgery. The subjective assessment of pain by patients on an analog scale in either group was minimal. No significant intra-operative complications were noted in either group. TetraVisc is superior to lidocaine 2% gel as topical anesthetic for patient comfort while preserving corneal clarity during surgery.

## Abbreviations

LG: Lidocaine gel; TV: Tetravisc; IOL: Intraocular lens.

## Competing interests

The authors declare that they have no competing interests.

## Authors' contributions

KVC and SG were involved in the conception and design of the study; SKG was involved in the acquisition of data; SA and RK were involved in the analysis of the data and preparation of the manuscript.

## Pre-publication history

The pre-publication history for this paper can be accessed here:


